# Recognition of mental disorders among the Singapore general population: an 8-year comparison

**DOI:** 10.1186/s12888-026-07784-w

**Published:** 2026-01-12

**Authors:** Shazana Shahwan, Bernard Chin Wee Tan, Yoke Boon Tan, Savita Gunasekaran, Wei Jie Ong, Saleha Shafie, Porsche Poh, Georg Schomerus, Siow Ann Chong, Mythily Subramaniam

**Affiliations:** 1 Research Division, Institute of Mental Health, Buangkok Green Medical Park, 10 Buangkok View, Singapore, 539747 Singapore; 2 Silver Ribbon, Singapore, 550208 Singapore; 3 Department of Psychiatry, University of Leipzig, 04103 Leipzig, Germany; 4 Department of Psychosis and East Region, Institute of Mental Health, Singapore, 539747 Singapore

**Keywords:** Mental health literacy, Recognition, Vignette, Public beliefs, Population study, Help-seeking

## Abstract

**Background:**

The ability to correctly recognise an acute mental health problem is an important aspect of mental health literacy (MHL). It is also a pre-requisite for prompt help-seeking. In Singapore, the treatment gap for mental disorders is large, with close to 80% who had not received help. Since the first MHL survey in 2015, significant advances have been made to raise mental health awareness. Thus, this study aims to examine the changes in MHL 8 years after the first survey and identify current correlates of MHL.

**Method:**

A vignette approach was used to assess recognition of five conditions, major depressive disorder (MDD), alcohol abuse, obsessive compulsive disorder (OCD), schizophrenia, and dementia. The analysis used data from participants who were randomly assigned one of these vignettes in the two Mind Matters nationwide surveys: 3,002 participants in 2023 and 3,006 participants in 2015. Logistic regression analysis was conducted to examine correlates of correct recognition in the 2023 sample.

**Results:**

Overall, recognition of the five conditions increased from 43.7 to 59.3%. Dementia, alcohol abuse and MDD remained the most recognised (≥ 65.4%). Recognition rates improved significantly for all the conditions except schizophrenia. The largest improvement was recorded for OCD with a 33.6% surge to 62.3%. Older age and lower education were associated with poorer dementia and schizophrenia recognition.

**Conclusions:**

MHL in Singapore increased since the 2015 study. However, there are still persistently low levels of literacy in relation to schizophrenia recognition and especially among those who are older and with lower educational attainment. Future mental health literacy strategies could include schizophrenia-specific content and culturally appropriate outreach to address these gaps.

**Clinical trial number:**

Not applicable.

**Supplementary Information:**

The online version contains supplementary material available at 10.1186/s12888-026-07784-w.

## Introduction

About one in every eight people in the world suffer from a mental illness [1] but only a minority of them seek professional help, often after a considerable period of delay [2]. A major barrier to help seeking is poor mental health literacy (MHL) [3]. The concept of MHL was first introduced in 1997 by Jorm and colleagues, defined by the authors as knowledge and beliefs about mental disorders which aid their recognition, management, and prevention [4]. Jorm and colleagues later refined the definition to include a person’s ability to put the acquired knowledge into actions for the benefit of their own health and that of the people around them [5].

The ability to correctly recognise and label an acute mental health problem and distinguish it from everyday problems is an important aspect of MHL [6]. The dismissal of mental illness symptoms as minor and insignificant have been highlighted in several studies [7, 8]. Respondents in these studies believed that they could manage the symptoms themselves or that the symptoms would go away on their own. Moreover, they did not want to ‘make a fuss’ for something that turns out to be ‘nothing’ and feared wasting a health professional’s time with such complaints [9].

The identification of mental illness by the public is a pre-requisite for prompt help-seeking for themselves or those around them suffering from the illness. Effective and timely treatment enhances the likelihood of a better prognosis of the illness. Good mental health knowledge is also associated with better self-awareness, adaptive health behaviours and therefore improved psychological well-being and outcomes [10, 11]. MHL can be improved at the level of the individual, community, and entire population – through an array of strategies including face-to-face interventions, national campaigns, or self-directed online channels among other means. Improvement in confidence of one’s ability to provide support to others with mental health issues have also resulted from interventions that enhance mental health knowledge [12, 13].

In Singapore, the treatment gap for mental illness is large despite it being considered a world leader of healthcare system efficiency for the past decade [14]. It has been a struggle to narrow this gap, with the Singapore Mental Health Study (SMHS) conducted first in 2010 and then 2016 both showing that more than three quarters of those with mental illnesses have not received treatment [15]. The Mind Matters 2015, a study of MHL, found that overall, less than half of the adult population in Singapore (43.7%) [16] could correctly recognise five selected mental health conditions, contributing to the large treatment gap. The literacy rates varied with some conditions being more well recognised (Alcohol Abuse: 57.1%) than others (Schizophrenia: 11.5%).

In the recent years, a raft of efforts to raise mental health awareness have been initiated such as the ‘Beyond the Label’ and ‘It’s OKAY to Reach Out’ national campaigns that were launched in 2018 and 2021, respectively. The ‘Mental Health Toolkit for employers’ was published in 2019 to guide employers in supporting persons with mental health conditions in workplaces. Mindline.sg, a one-stop digital mental health resource portal was launched in 2020 and the ‘Do You M.I.N.D’, an experiential learning programme using virtual technology was brought to secondary schools to educate young people on conditions like eating disorders, depression, anxiety and self-harm, to name a few of these efforts. Singapore’s National Mental Health and Well-being Strategy was launched in 2023 with the aim of creating an effective mental health ecosystem comprising accessible and quality care, and a supportive society. In 2024, the nation’s then-Deputy Prime Minister announced mental health as a key priority in Singapore’s national agenda [17].

Changing trends in the population such as higher levels of education, income, access to digital information, and shifts in mindsets and social norms (e.g. self-development movements) may also effect a change in MHL [18–20]. It is therefore important to track trends in MHL in the resident population in order to identify current areas of need that limited resources can be pragmatically allocated to. The present study launched in 2023 aimed to examine changes in MHL, operationalised as correct recognition of five selected conditions – major depressive disorder, alcohol abuse, obsessive-compulsive disorder (OCD), schizophrenia and dementia – since the first Mind Matters study conducted in 2015. These disorders were selected based on a number of factors which were established by surveys on the mental health status of the adult and older adult population of Singapore [21–24], including their relatively high prevalence in the population, and in particular, the large treatment gap of each of these disorders [15, 25]. The second aim was to identify correlates of literacy to inform targets for future interventions. It is hypothesized that MHL would increase in the 2023 survey compared to the 2015 survey.

## Method

### Participants

4195 Singapore Citizens and Permanent residents aged 18–67 were recruited during the second Mind Matters cross-sectional survey, also known as the Mind Matters 2023. The survey fieldwork period lasted from September 2022 to February 2024. The sampling frame was based on an updated administrative database of all Singapore citizens and permanent residents. A probability sample was randomly selected using a disproportionate stratified design defined according to ethnicity (Chinese, Malay, Indian and Other) and age group (18–34, 35–49, 50–65). A detailed description of the study design is reported in Subramaniam, et al. [26]. The study yielded a response rate of 62%. Data from 3002 participants who were assigned any one of the five vignettes (i.e. major depressive disorder, alcohol abuse, OCD, schizophrenia and dementia) which were also used in the 2015 study, were included in the current analysis for comparisons. Data from the remaining participants (*n* = 1193) who were assigned the two new vignettes, gambling disorder and depression with suicidality introduced only in the 2023 study were excluded and described in separate publications [27, 28].

Participants in the first study conducted between March 2014 and April 2015 (i.e. Mind Matters 2015) comprised 3006 residents who were sampled using an identical methodology. The response rate for this earlier study was 71%. Detailed description of this first study is reported in Chong, et al. [16]. The sociodemographic distribution of the Mind Matters 2015 and 2023 samples is presented in Table 1.

**Table 1:** Sociodemographic distribution of the Mind Matters 2015 and 2023 samples

Sociodemographic variables	Mind Matters 2015 (n = 3006)		Mind Matters 2023 (n = 3002)		
	*N*	Weighted %	*N*	Weighted %	*p* value
Age groups					
18–34	1152	34.4	984	31.5	
35–49	896	35.2	949	31.6	
50–67	958	30.5	1069	37.0	**< 0.001**
Gender					
Female	1506	49.1	1534	50.9	
Male	1500	50.9	1468	49.1	0.317
Ethnicity					
Chinese	1034	74.7	822	73.8	
Malay	977	12.8	957	13.2	
Indian	963	9.1	948	9.3	
Others	32	3.3	275	3.7	0.829
Marital status					
Never Married	927	31.4	928	33.4	
Currently married	1916	64.0	1865	59.8	
Separated/ Divorced/ Widowed	162	4.6	209	6.8	**0.008**
Highest education					
Primary and below	431	13.4	235	7.5	
Secondary/ O Levels/ N Levels	820	25.8	684	20.9	
A Level/ Polytechnic Diploma/ Other Diploma	999	31.3	1035	30.3	
University and above	756	29.6	1048	41.3	**< 0.001**
Employment status					
Currently employed	2227	77.6	2376	81.1	
Unemployed	120	3.9	124	3.7	
Economically inactive	659	18.4	502	15.2	**0.032**
Monthly personal income					
Below 2,000	1346	40.5	1023	31.6	
2000 to 5999	1162	46.4	1374	44.9	
6000 and above	294	13.1	567	23.5	**< 0.001**

### Procedure

Invitation letters describing the purpose and procedure of the study were sent by post to the randomly selected residents. They were then visited at their homes by a trained lay interviewer. Prior to commencement of any study procedures, the interviewer explained the study and obtained written informed consent from participants (and their legally acceptable representative for respondents under 21 years of age, the age of majority in Singapore). The survey was administered by interviewers using a tablet. Consent-taking and the survey were offered in English, Mandarin, Malay, or Tamil, the four official languages. The survey took about 1.5 h and participants were compensated S$40 as an inconvenience fee for their time and effort. The study was approved by the National Healthcare Group Domain Specific Review Board.

### Measures

Sociodemographic information: Age, gender, ethnicity, highest level of educational attainment, marital and employment status as well as income was collected.

Vignette: The five vignettes previously developed for the Mind Matters 2015 were used. These vignettes were developed and revised in consultation with experienced research psychiatrists and further vetted by a panel of senior psychiatrists to ensure that the symptoms described satisfied the DSM-IV diagnostic criteria for each condition. The vignettes were then tested using cognitive interviews to ensure that the language was easily and correctly understood by the lay population [16].

Only one vignette was assigned to each respondent via a randomisation algorithm. The names of the fictional character (XX) described in the vignette were matched to the gender and ethnicity of respondent to facilitate identification and reduce risk of potential biases (e.g. Mr Tan for a Chinese, male and Siti for a Malay, female respondent). This was programmed into the computer-assisted personal interview application. The vignettes are included in Supplementary File 1.

Recognition of mental health condition: Participants were asked an open-ended question as follow up to the vignette: ‘What do you think XX is suffering from?’. Responses were coded as (i) ‘Correct Recognition’ (where the correct medical label or close approximations were assigned), (ii) ‘Recognise as a mental disorder’ (in instances of mislabelling with other mental illnesses or identification as mental illness without specification) or (iii) ‘Did not recognise as a mental disorder’ (for any other responses including stating of symptoms or labelling the condition as stress or ‘don’t know’). Each vignette was coded by two researchers with, at minimum, a post-graduate degree in Psychology, and guided by the codebook used in the 2015 study. Discrepancies for responses which had not been previously identified in the 2015 study were resolved through consultation with a senior consultant psychiatrist in the team (CSA) and lead researcher (MS). The analysis in the current paper focuses on ‘Correct Recognition’.

Personal experiences with mental illness: Participants were asked close-ended questions, ‘Have you ever had problems similar to XX’s?’, ‘Have you ever had a job that involved providing treatment or mental health services to a person with a problem like XX?’ and ‘Has anyone in your family or close circle of friends ever had problems similar to XX?’. Responses to these questions were ‘Yes’, ‘No’, ‘Don’t know’ or ‘Refuse to answer’. ‘Don’t know’ and ‘Refuse to answer’ responses were treated as missing data and were excluded from analysis of the item.

### Statistical analysis

All statistical analyses were carried out using SAS Version 9.4. Descriptive statistics were used to discern the sociodemographic characteristics for both the 2015 and 2023 study population. To ensure that the findings were representative of the Singapore population, estimates were weighted to adjust for the disproportionate sampling technique and post-stratified for age group and ethnicity distributions based on the 2015 and 2023 Singapore Population Census respectively. To further address non-response, propensity scores (estimating non-response likelihood) were calculated and included into the final weights, with respondents resembling the profile of non-responders in terms of age, gender, and ethnicity, having higher weights. Correct recognition was dichotomised (correct recognition vs. mislabelled/no recognition) for the analysis.

Chi-square tests were run to examine differences in the proportion of correct recognition overall and for each vignette between 2015 and 2023. Multiple logistic regressions were run to examine the associations of having prior personal contact (had problems themselves, had a job providing treatment, and had a friend or family member with similar conditions), and sociodemographic correlates with correct recognition for each of the mental health conditions. Sociodemographic correlates include age groups, gender, ethnicity, employment status, highest educational attainment, marital status, and monthly personal income.

## Results

**Fig. 1:**
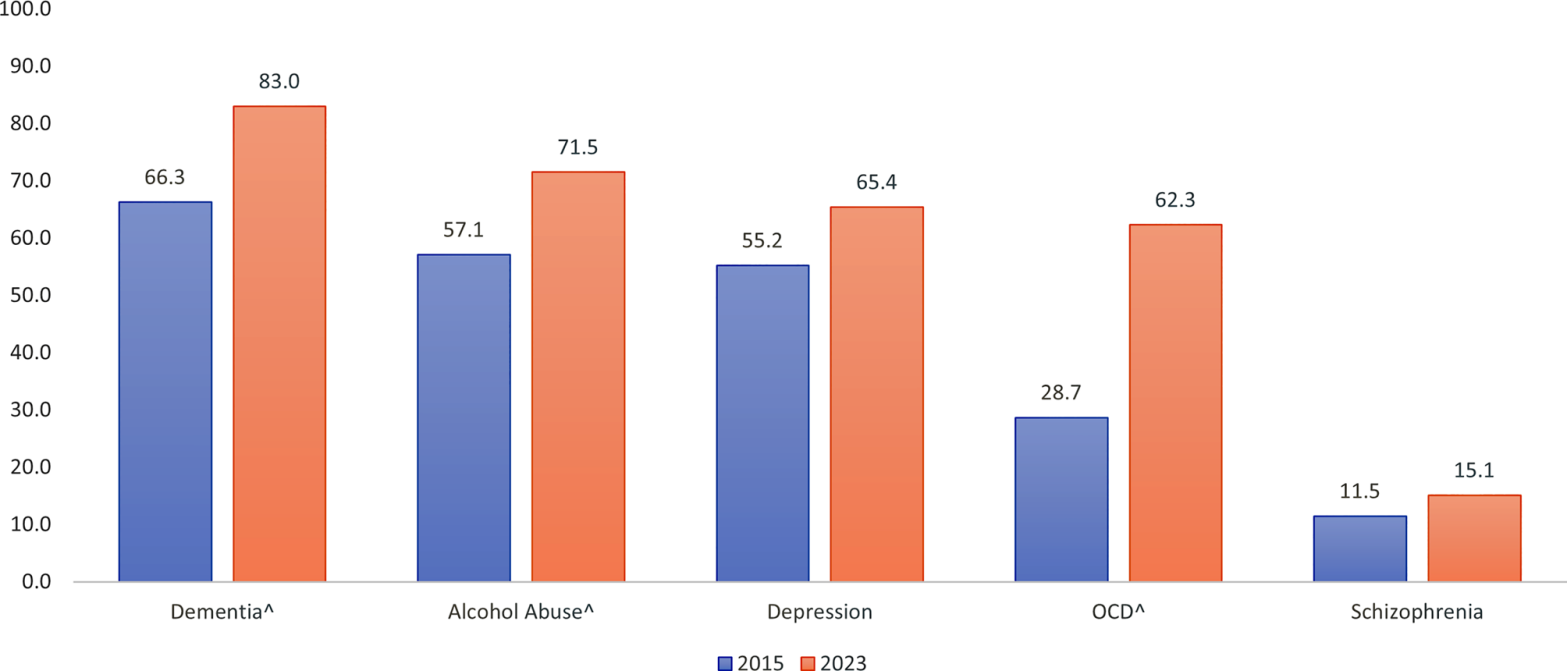
Comparison of the percentages between the 2015 and 2023 samples that correctly recognised the 5 mental health conditions. ^ *p* < 0.01,^+^ *p* < 0.05; chi-square comparisons were used to determine significance

The overall recognition of the 5 conditions significantly increased from 43.7% to 59.3% (*p* < 0.001). Significant increases were observed for all conditions except schizophrenia which saw an increase of only 3.6%. Dementia (83.0% vs. 66.3%, *p* < 0.001), Alcohol Abuse (71.5% vs. 57.1%, *p* < 0.001) and Depression (65.4% vs. 55.2%, *p* = 0.011) remained the most recognised conditions while OCD (62.3% vs. 28.7%, *p* < 0.001) and Schizophrenia (15.1% vs. 11.5%, *p* = .192) were the least recognised conditions. However, the sharpest improvement was seen for OCD with an improvement of 33.6% (Fig. 1).

### Sociodemographic correlates

Logistic regression models (Table 2) showed that individuals in the 35–49 age group (vs. 18–34, OR = 0.40, *p* = 0.045), of Indian ethnicity (vs. Chinese, OR = 0.40, *p* = 0.004), having a primary education or below (vs. University, OR = 0.23, *p* = 0.022) were less likely to recognise dementia while students (vs. currently working, OR = 5.42, *p* = 0.014) and those who had an income of SGD2000-5999 (vs. < SGD2000, OR = 2.78, *p* = 0.026) were more likely to recognise it. Females (vs. males, OR = 2.33, *p* = 0.006), Malays (vs. Chinese, OR = 2.33, *p* = 0.006), students (vs. currently working, OR = 15.04, *p* = 0.008) and those earning more than SGD6000 (vs. < SGD2000, OR = 3.53, *p* = 0.026) were more likely to recognise OCD. Individuals aged 35–49 and 50–65 (vs. 18–34, OR = 0.32, *p* = 0.01 and OR = 0.29, *p* = 0.045 respectively) and those with primary education or below (vs. university, OR = 0.04, *p* = 0.020) were less likely to recognise schizophrenia while those who were unemployed (vs. currently working, OR = 6.73, *p* = 0.015) were more likely to be able to recognise it. Those of ‘Other’ ethnicity (vs. Chinese, OR = 2.85, *p* = 0.033) were more likely to recognise alcohol abuse while those who had been separated, widowed, or divorced (vs. currently married, OR = 0.21, *p* = 0.003) were less likely to recognise it. None of the sociodemographic variables were associated with depression.

**Table 2:** Sociodemographic correlates of correct recognition of mental health conditions by vignettes in the 2023 sample

		Dementia				Alcohol Abuse				Depression				OCD				Schizophrenia			
		OR	95% CI		*p* value	OR	95% CI		*p* value	OR	95% CI		*p* value	OR	95% CI		*p* value	OR	95% CI		*p* value
Age groups	18–34	Reference group																			
	35–49	0.40	0.16	0.98	**0.045**	1.12	0.50	2.50	0.783	0.67	0.30	1.48	0.318	0.87	0.38	2.00	0.745	0.32	0.14	0.76	**0.010**
	50–67	1.13	0.39	3.23	0.826	1.01	0.45	2.30	0.975	0.57	0.25	1.28	0.172	0.47	0.19	1.15	0.097	0.29	0.08	0.98	**0.045**
Gender	Male	Reference group																			
	Female	0.92	0.47	1.81	0.816	0.94	0.52	1.72	0.848	1.337	0.783	2.281	0.287	2.33	1.273	4.247	**0.006**	1.749	0.830	3.685	0.141
Ethnicity	Chinese	Reference group																			
	Indian	0.40	0.22	0.74	**0.004**	0.83	0.50	1.39	0.475	0.861	0.526	1.407	0.549	1.35	0.82	2.24	0.241	0.83	0.42	1.62	0.581
	Malay	0.64	0.33	1.22	0.174	0.85	0.49	1.48	0.554	0.957	0.573	1.600	0.868	2.09	1.20	3.63	**0.009**	0.94	0.43	2.07	0.885
	Others	0.62	0.26	1.53	0.304	2.85	1.09	7.42	**0.033**	0.941	0.445	1.990	0.874	1.24	0.62	2.51	0.541	0.47	0.13	1.71	0.253
Marital Status	Currently Married	Reference group																			
	Never Married	0.89	0.37	2.14	0.789	1.38	0.65	2.95	0.407	1.32	0.60	2.94	0.49	1.39	0.61	3.18	0.437	1.31	0.62	2.76	0.481
	Separated/ Widowed/ Divorced	0.44	0.17	1.13	0.089	0.21	0.07	0.58	**0.003**	1.07	0.41	2.75	0.90	0.76	0.29	2.01	0.580	2.90	0.61	13.73	0.180
Education	Primary and below	0.23	0.06	0.81	**0.022**	1.49	0.41	5.48	0.547	0.73	0.22	2.45	0.61	0.39	0.11	1.42	0.153	0.04	0.00	0.61	**0.021**
	Secondary/ O Levels/ N Levels	0.75	0.26	2.11	0.579	0.62	0.26	1.47	0.274	0.66	0.29	1.51	0.33	0.90	0.40	2.07	0.812	0.51	0.14	1.96	0.330
	A Level/ Polytechnic Diploma/ Other Diploma/ ITE Certificate	0.85	0.31	2.32	0.755	0.92	0.43	1.95	0.825	0.99	0.48	2.02	0.970	0.9	0.4	1.8	0.708	0.98	0.42	2.31	0.963
	University	Reference group																			
Employment status	Currently working	Reference group																			
	Housewife/ husband	1.80	0.42	7.77	0.433	0.71	0.22	2.27	0.566	1.95	0.60	6.32	0.263	2.13	0.60	7.60	0.243	0.52	0.09	3.17	0.480
	Retired	2.04	0.50	8.31	0.318	4.22	0.97	18.32	0.054	0.63	0.09	4.56	0.649	3.87	0.86	17.41	0.078	5.26	0.70	39.48	0.107
	Student	5.42	1.40	20.99	**0.014**	3.52	0.64	19.28	0.146	1.00	0.22	4.58	0.997	15.04	2.02	112.19	**0.008**	0.69	0.14	3.42	0.647
	Unemployed	0.91	0.25	3.35	0.887	0.43	0.11	1.65	0.219	2.87	0.64	12.88	0.168	1.28	0.38	4.35	0.746	6.73	1.45	31.15	**0.015**
Personal Income	Below 2000	Reference group																			
	2000–5999	2.78	1.13	6.83	**0.026**	1.08	0.50	2.32	0.845	1.91	0.88	4.15	0.103	2.38	1.00	5.70	0.051	0.47	0.16	1.35	0.158
	6000 and above	4.00	0.91	17.56	0.066	1.73	0.58	5.18	0.327	1.40	0.50	3.94	0.528	3.53	1.16	10.75	**0.026**	1.95	0.63	6.00	0.247

### Contact-related experiences

Logistic regression models (Table 3) showed that those who had a job providing treatment for individuals with dementia (OR = 3.61, *p* = .029) or had family members or friends with the condition (OR = 4.15, *p* = .001) were more likely to be able to recognise it. Individuals who had problems like the person in the schizophrenia vignette (OR = 0.06, *p* = .024) were less likely to be able to correctly recognise it, in contrast to someone who had a job providing treatment for this condition (OR = 6.40, *p* = .009).

**Table 3:** Personal contact-related correlates of correct recognition by vignettes

		Dementia				Alcohol Abuse				Depression				OCD				Schizophrenia			
		OR	95% CI		*p* value	OR	95% CI		*p* value	OR	95% CI		*p* value	OR	95% CI		*p* value	OR	95% CI		*p* value
Self had problems^1^	Yes	0.57	0.22	1.46	0.241	0.57	0.21	1.53	0.265	1.82	1.00	3.33	0.051	1.72	0.66	4.48	0.264	0.06	0.01	0.68	**0.024**
	No	Reference group																			
Had job providing treatment^2^	Yes	3.61	1.14	11.43	**0.029**	0.94	0.24	3.67	0.933	3.16	0.97	10.28	0.056	0.71	0.26	1.94	0.498	6.40	1.61	25.48	**0.009**
	No																				
Family or friends with similar problems^3^	Yes	4.15	1.77	9.73	**0.001**	0.52	0.27	1.02	0.057	0.88	0.49	1.60	0.673	0.90	0.42	1.92	0.787	2.82	0.99	8.05	0.052
	No																				

^1^ *N* = 3 responded as don’t know/refused and were recoded to missing, ^2^ *N* = 7 responded as don’t know/refused and were recoded to missing, ^3^ *N* = 7 responded as don’t know/refused and were recoded to missing

### Post-hoc analysis

A post-hoc analysis was carried out to investigate a possible explanation for lower Alcohol Abuse recognition rates among those who were ‘Separated, widowed or divorced’. Specifically, associations between survey items relating to personal experiences with Alcohol Abuse and being ‘Separated widowed or divorced’ were examined. Chi square analysis revealed that a higher proportion of individuals who were separated, widowed, or divorced had problems similar to the character in the alcohol abuse vignette (32.4%) compared to those who were never married (11.7%) or currently married (4.2%) (*p* = .002) with logistic regression analysis providing support that the differences were statistically significant. Individuals who were currently married (OR: 0.09, CI: 0.02–0.35, *p* < .001) and never married (OR: 0.28, CI:0.77–0.995, *p* < .049) were significantly less likely to have had problems similar to the character in the alcohol abuse vignette than those who were separated, divorced or widowed.

## Discussion

MHL in Singapore increased in 2023 compared to 2015 in line with our hypothesis. Overall, correct recognition of the five selected conditions rose significantly from 43.7% to 59.3%. This trend is in keeping with those observed in other industrialised countries [29–31] following the implementation of national mental health awareness campaigns. The top three most recognised conditions were dementia, alcohol abuse and depression, as with the first Mind Matters 2015 survey. The slew of local dementia-related initiatives and extensive portrayal of alcohol abuse and depression in popular media which were offered as likely reasons for the comparatively better recognition rates in the earlier survey may have persisted [16], and the recent awareness efforts could have resulted in further improvements.

OCD remained the second least recognised condition. Improvement in its recognition, however, was the most striking, with an upsurge of 33.6% to 62.3% – more than doubling the percentage in the 2015 study of only 28.7%. This surge could be due to several factors in addition to the awareness efforts. A study found that OCD symptoms increased in non-clinical populations during the COVID-19 outbreak which may have led to information-seeking about the condition [32]. Stigma towards this condition was also noted to be lower in comparison to other conditions [26]. It is of concern still, that several studies have reported on the widespread flippant use of the term OCD in social media in the recent years. For instance, the hashtag #OCD is used as a shorthand to mockingly label someone who does not like untidiness [33]. While the increase in recognition could reflect actual improvement in literacy, the levity with which the diagnostic term is used is worrying as it trivialises the experience of those besieged by the condition. The trivialisation of the illness was an issue earlier identified in a local focus group discussion among those with lived experience [34], which could inhibit treatment-seeking. Thus, future awareness campaigns can bring to light the harms of such oversimplification. Advocates on social media are particularly influential in presenting the illness with authenticity and accuracy [33]. Future surveys could examine if the improved recognition has translated to narrowing the wide OCD treatment gap of 84% reported in the SMHS 2016 [15], and examine nuances in perceptions regarding OCD as well as how it relates to attitudes towards obtaining professional advice.

In contrast to OCD, recognition for schizophrenia remained low. It was the only condition for which the increase in recognition was non-significant. The rate of schizophrenia recognition rate in the Singapore’s general population is lower than that of other Western countries. In Germany, correct labelling of schizophrenia increased from 17.5% in their 1990/1993 study compared to 34.3% in 2020. While depression was more well-recognised in their sample as well, the contrast between the two conditions was not as sharp as the current study, with the increase in depression recognition being 26.5% to 46.3% between 1990/1993 and 2020. Recognition rate for schizophrenia in Australia was 37.3% among the general population in 2011 [35]. In China, recognition of schizophrenia among caregivers of patients with mental disorder too was surprisingly low at 28.5% compared to depression which was 43.6% [36]. One reason why schizophrenia recognition did not increase with the rest of the conditions could be the focus of local mental health efforts on emotional disorders of higher prevalence such as depression and anxiety while schizophrenia received less coverage. Another challenge to improving understanding of the condition relates to negative media portrayal where schizophrenia is often associated with violence and dangerousness [37, 38]. Thus, it is not surprising that stigma and desire for social distance towards this condition is high [26]. Furthermore, in South-east Asian cultures, psychotic symptoms are often believed to be disturbances induced by black magic or spirit possession [39] which could result in information- or help-seeking from traditional sources. There is also a possibility that the schizophrenia vignette, without contextual details like common age of onset while describing the character as living in a single-room public housing, may have resulted in misattribution of symptoms to an outcome of a life of solitude or isolation. Additionally, ‘incorrect’ responses may represent a culturally-influenced way of reframing the condition to avoid a stigmatising label. To illustrate this point, Yang, et al. demonstrated that a vignette describing a person with schizophrenia labelled with the cultural idiom of “excessive thinking (*xiang tai duo*)” elicited the least desire for social distance compared to the same vignettes that were labelled with ‘schizophrenia’ indicating that cultural idioms provide some protection against stigma [40]. However, using alternative labels to limit the impact of stigma can contribute to the ‘conspiracy of silence’ with participants not knowing their diagnosis and maintaining a lack of understanding of their condition [41]. Due to the severity and chronicity of this disorder and the immense impact on the individual, family, and society, it is important to raise awareness that these symptoms could be due to schizophrenia and that effective medical treatments are available.

It was observed that MHL differed by sociodemographic lines in the population. Those in the older age groups of 35–49 or 35–67 years (versus those aged 18–34) were less likely to be able to recognise dementia and schizophrenia respectively. Those who had received the least amount of formal education were also less able to recognise these conditions compared to those who have attended university. Older age and lower educational attainment have been associated with lower MHL in the earlier studies [42, 43]. This is because many programmes are often delivered in schools and institutions or delivered through media which may be less commonly accessed by these groups [42]. It is of note that those in the 35–49 age group and of Indian ethnicity were associated with the least dementia recognition. As those in the 40–59 age group make up 60% of dementia caregivers in Singapore [44] and Indian ethnicity has been identified to have higher dementia risk in earlier local epidemiological research [24, 45], further improving dementia awareness such as correcting the common misperception that dementia symptoms are a part of normal ageing [43, 46] and sharing treatment avenues among these groups, can facilitate provision of support and alleviate caregiver burden [47].

Being separated, widowed, or divorced was associated with lower recognition of alcohol abuse. Those who were in this demographic tended to endorse having problems depicted in the alcohol abuse vignette. Denial which is a cardinal characteristic in addictive disorders could explain the low recognition in this group [48]. Individuals who have undergone marital separation often cite substance use as having contributed to their marriages dissolution with a study that interviewed 52 divorcees reporting substance use to be among the most common ‘final straw’ reasons, alongside infidelity and domestic violence [49]. Of concern, the treatment gap for alcohol dependence reported in the SMHS 2016 was the highest compared to other conditions where 97% of those who met criteria did not receive any treatment [15]. The authors cited high stigma and public perception of alcoholism as a moral failure as barriers to accessing treatment. Sensitive messaging to reduce blame and render help to families with alcohol use problems are needed. Studies have found that marital and family therapies are effective in helping the family cope better and in motivating the family member with alcohol-related problems to enter treatment [50].

On the other hand, other demographic factors were associated with better recognition of mental health conditions. In particular, females and those of Malay ethnicity had better recognition of OCD while ‘Other ethnicity’ was associated with better recognition of Alcohol Abuse. While reasons for these associations have not been investigated, the pattern of findings are reflective of the elevated prevalence rate of OCD in females and Malays, and drinking patterns among those in the Other ethnic group (which in Singapore are residents with Eurasian or Caucasian origins though other ethnicities like Japanese or Thai may also fall in this group) [51, 52]. Another possible reason for the gender difference in OCD recognition was demonstrated by Stewart, et al. where males were more likely to describe OCD as perfectionism and did not see a difference between someone who was obsessive-compulsive and having OCD [53]. Future studies can examine the impact of improved recognition in some demographic groups compared to others on their help-seeking behaviours.

Of note, unlike the Mind Matters 2015 study in which males, Indian ethnicity and having primary level education or less were associated with poor recognition of depression, these differences were no longer significant in the current study. This change could represent the narrowing of social inequality of depression literacy. Currently, moving a step further, preventive efforts are being carried out to aid detection of subsyndromal depression [54] or mild to moderate psychological problems [55] and offer care in the community settings to prevent progression to clinically relevant levels with the aim of reducing the individual’s distress and preserving quality of life as well as productivity.

Next, in line with Allport’s contact hypothesis [56] that personal contact with a person with mental illness can lead to better knowledge and understanding towards them, individuals who ever had a job providing treatment for patients with dementia and schizophrenia and those with family or friends with dementia were more likely to be able to label these vignettes correctly. Professional training from the job and efforts to learn more about the conditions of loved ones are likely to enhance recognition. By contrast, those who had experienced schizophrenia symptoms were less likely to recognise it. Insight, which refers to awareness of having a mental health condition, the ability to attribute delusions and hallucinations to a mental illness and recognition of the need for medication, has been shown to be at least partly lacking in a large proportion of patients with schizophrenia [57]. As such, equipping family member and friends with knowledge on the condition and its treatment is an essential part to facilitating better outcomes, prognosis and functional recovery of the individual with the condition.

### Implications

Overall, the findings highlight significant improvements in recognition of mental health conditions which follow the wave of efforts for mental health awareness and the government’s efforts to make it a national priority amidst other advances such as wider access to online information, greater literacy rates in general and Gross Domestic Product (GDP) growth since the first Mind Matters survey in 2015 [58]. The current study points to gaps that need to be addressed, particularly for schizophrenia and poorer literacy among those who are older and with lower education. In a review highlighting initiatives to reduce stigma towards individuals with complex mental disorders in Australia, some strategies could be adapted to the Singapore context. Campaigns targeted specifically towards schizophrenia could be organised such as Schizophrenia Awareness week which includes sharing of lived experience stories, with an emphasis on recovery and hope and spreading messages to dispel myths and negative stereotypes surrounding schizophrenia. Online resources accessible to the public with notable reach such as episodes on ‘You can’t ask that’ or ‘The feed’ produced by Australia’s national broadcasters featured persons with schizophrenia, providing insight to living with the condition and addressing public misconception. These episodes attained high viewership [13]. Culturally appropriate approaches were also developed for Australia’s Macedonian community where a theatre play about a family with a son with schizophrenia, reached 1600 people in this community. Such culturally informed strategies can be adopted to reach out to communities in Singapore that may not attend formal institutions or frequent social media platforms. These strategies may include community engagement carried out in mother tongue or dialect languages and holding roadshows at venues where the subgroups congregate such as neighbourhood malls, community centres or even places of worship, and spreading mental health messages through cultural arts-based performances or other social activities. Lastly, contact-based approaches that have been promising in improving attitudes towards mental health conditions can be incorporated into these interventions [59, 60].

## Strengths and limitations

The strengths of the study are the use of a vignette-based approach developed by local psychiatrists and researchers and cognitively tested to ensure that that they were appropriate to the local context. These were offered in the local languages preferred by the respondent to ensure inclusivity and generalisability to the Singapore population. Next, the study used a methodology that was similar to the first Mind Matters study that allows for a direct comparison and tracking of recognition in the population. The study yielded a reasonable response rate and included a large representative sample of the general population. However, the findings should be interpreted in light of the following limitations. First, there was a non-response rate of 38%, higher than the first study. Although sample weights based on age, gender and ethnicity were applied to adjust for non-response, one of the reasons stated by residents for not responding was that they did not want to participate in a survey associated with mental health [26]. Reasons for non-response such as this may be associated with lack of interest or confidence in answering questions on mental health knowledge which may have affected the overall results. Additionally, the 2023 sample had higher levels of education, employment and income which has been shown to be correlated with higher mental health literacy in previous studies [20, 61]. While improvements in these socioeconomic indicators are reflective of the Singapore population census trends [62, 63], we are not able to rule out the possibility that the higher recognition rates observed in this study could have been inflated due to self-selection bias. The study did not account for demographic or societal changes in Singapore between 2015 and 2023 such as population and economic growth. Future research that takes these societal-level indicators into account could provide a more comprehensive picture of changes in mental health knowledge over time. Second, as the study is cross-sectional in nature, it is unable to delineate the factors that resulted in the changes between the two studies and thirdly, recognition captures only one aspect of MHL. Being able to name an illness correctly facilitates discussion of the condition with a common parlance. However, it is possible for incorrect responses to be due to reasons other than lack of awareness– such as to manage stigma. In the current survey, gender and ethnicity of the character in the vignette was matched to the participant to reduce the influence of stereotypes. Notwithstanding, stereotypes and gender role expectations could have still influenced the results as several studies have shown that respondents were more likely to rate a female or feminine character as suffering from a mental disorder than a male or masculine character [53, 64, 65]. In keeping the vignettes succinct, details that may have aided recognition were omitted. As such, future studies could examine the role that sociodemographic variations and contextual descriptors play in recognition of mental health conditions. While the Mind Matters 2023 survey examined other aspects of mental health literacy which is beyond the scope of this paper such as stigma, help-seeking and causal attributions, which also pointed to improvement in MHL over the years [26, 28, 66] the survey did not include other measures of MHL such as knowledge on how to support someone with a mental health problem. Covering multiple domains of MHL can be considered in future studies to provide a more comprehensive evaluation of the MHL construct [67].

## Supplementary Information

Below is the link to the electronic supplementary material.

Supplementary Material 1

## Data Availability

The datasets generated and/or analysed during the current study are not publicly available due institutional policy but are available from the corresponding author on reasonable request.

## References

[1] WHO. Mental disorders. 2022 [cited 2025 Jun 21]. Available from: https://www.who.int/news-room/fact-sheets/detail/mental-disorders.

[2] Osman N, Michel C, Schimmelmann BG, Schilbach L, Meisenzahl E, Schultze-Lutter F. Influence of mental health literacy on help-seeking behaviour for mental health problems in the Swiss young adult community: a cohort and longitudinal case–control study. Eur Arch Psychiatry Clin Neurosci. 2023 Apr 1 [cited 2025 Jun 21];273(3):649–62. Available from: https://pubmed.ncbi.nlm.nih.gov/36088495/. 10.1007/s00406-022-01483-9 PMC10085901 36088495

[3] Kelly CM, Jorm AF, Wright A. Improving mental health literacy as a strategy to facilitate early intervention for mental disorders. Med J Aust. 2007 [cited 2025 Jun 21];187(7 Suppl). Available from: https://pubmed.ncbi.nlm.nih.gov/17908021/. 10.5694/j.1326-5377.2007.tb01332.x 17908021

[4] Jorm AF, Korten AE, Jacomb PA, Christensen H, Rodgers B, Pollitt P. Mental health literacy: A survey of the public’s ability to recognise mental disorders and their beliefs about the effectiveness of treatment. Med J Aust. 1997 Feb 17 [cited 2025 Jun 21];166(4):182–6. Available from: https://pubmed.ncbi.nlm.nih.gov/9066546/. 10.5694/j.1326-5377.1997.tb140071.x 9066546

[5] Jorm AF. The concept of mental health literacy. Int Handb Health Lit. 2019;53–66.

[6] Grohmann E, Al-Addous A, Sander C, Dogan-Sander E, Baumann E, Angermeyer MC, et al. Changes in the ability to correctly identify schizophrenia and depression: results from general population surveys in Germany over 30 years. Soc Psychiatry Psychiatr Epidemiol. 2024 Oct 1 [cited 2025 Jun 21];59(10). Available from: https://pubmed.ncbi.nlm.nih.gov/38583103/. 10.1007/s00127-024-02660-y PMC11481663 38583103

[7] Smith JA, Braunack-Mayer A, Wittert G, Warin M. It’s sort of like being a detective: Understanding how Australian men self-monitor their health prior to seeking help. BMC Health Serv Res. 2008 Mar 14 [cited 2025 Jun 21];8(1):1–10. Available from: https://bmchealthservres.biomedcentral.com/articles/10.1186/1472-6963-8-56. 10.1186/1472-6963-8-56 PMC2288603 18366631

[8] Smith JA, Braunack-Mayer AJ, Wittert GA, Warin MJ. Qualities men value when communicating with general practitioners: Implications for primary care settings. Med J Aust. 2008 Dec 15 [cited 2025 Jun 21];189(11–12):618–21. Available from: https://pubmed.ncbi.nlm.nih.gov/19061448/. 10.5694/j.1326-5377.2008.tb02214.x 19061448

[9] O’Brien R, Hunt K, Hart G. It’s caveman stuff, but that is to a certain extent how guys still operate: Men’s accounts of masculinity and help seeking. Soc Sci Med. 2005 Aug [cited 2025 Jun 21];61(3):503–16. Available from: https://pubmed.ncbi.nlm.nih.gov/15899311/. 10.1016/j.socscimed.2004.12.008 15899311

[10] Hahn JS, Chua KC, Jones R, Henderson C. The Every Mind Matters campaign: changes in mental health literacy and its associations with campaign awareness. Eur J Public Health. 2023 Dec 1 [cited 2025 Jun 21];33(6):1008–13. Available from: https://pubmed.ncbi.nlm.nih.gov/37579223/. 10.1093/eurpub/ckad145 PMC10710357 37579223

[11] Schomerus G, Stolzenburg S, Freitag S, Speerforck S, Janowitz D, Evans-Lacko S, et al. Stigma as a barrier to recognizing personal mental illness and seeking help: a prospective study among untreated persons with mental illness. Eur Arch Psychiatry Clin Neurosci. 2019 Jun 1 [cited 2025 Jun 21];269(4):469–79. Available from: https://pubmed.ncbi.nlm.nih.gov/29679153/. 10.1007/s00406-018-0896-0 29679153

[12] Brijnath B, Protheroe J, Mahtani KR, Antoniades J. Do web-based mental health literacy interventions improve the mental health literacy of adult consumers? results from a systematic review. J Med Internet Res. 2016 Jun 1 [cited 2025 Jun 21];18(6). Available from: https://pubmed.ncbi.nlm.nih.gov/27323907/. 10.2196/jmir.5463 PMC4932246 27323907

[13] Morgan AJ, Wright J, Reavley NJ. Review of Australian initiatives to reduce stigma towards people with complex mental illness: what exists and what works? Int J Ment Health Syst. 2021 Dec 1 [cited 2025 Jun 21];15(1):1–51. Available from: https://ijmhs.biomedcentral.com/articles/10.1186/s13033-020-00423-1. 10.1186/s13033-020-00423-1 PMC7814561 33461567

[14] Szücs A, Teo DCL, De La Arias J, Subramaniam M, Valderas JM. Integrating mental health care into primary and community care in Singapore: a vision based on Healthier SG. Lancet Reg Health West Pac. 2025 Jan 1 [cited 2025 Jun 21];54:101279. Available from: https://www.thelancet.com/action/showFullText?pii=S2666606524002736. 10.1016/j.lanwpc.2024.101279 PMC11751500 39845983

[15] Subramaniam M, Abdin E, Vaingankar JA, Shafie S, Chua HC, Tan WM, et al. Minding the treatment gap: results of the Singapore Mental Health Study. Soc Psychiatry Psychiatr Epidemiol. 2020 Nov 1 [cited 2025 Jun 21];55(11):1415–24. Available from: https://pubmed.ncbi.nlm.nih.gov/31317246/. 10.1007/s00127-019-01748-0 PMC7578124 31317246

[16] Chong SA, Abdin E, Picco L, Pang S, Jeyagurunathan A, Vaingankar JA, et al. Recognition of mental disorders among a multiracial population in Southeast Asia. BMC Psychiatry. 2016;16(1). 10.1186/s12888-016-0837-2 PMC4855433 27142577

[17] Agarwal Chirag. The Straits Times. 2025 [cited 2025 Jun 21]. Mental health is now on the national agenda – let’s cut to the chase | The Straits Times. Available from: https://www.straitstimes.com/opinion/mental-health-is-now-on-the-national-agenda-lets-cut-to-the-chase.

[18] Pan J, Xu T, Li D. The relationship between mental health literacy and social well-being: a longitudinal study in China. Behav Sci. 2024 Dec 30 [cited 2025 Dec 22];15(1):29. Available from: https://www.mdpi.com/2076-328X/15/1/29/htm. 10.3390/bs15010029 PMC11763095 39851833

[19] Wu J, Shen H, Shen Y, Liao X, Yu X. The influence of family socioeconomic status on college students’ mental health literacy: the chain mediating effect of parenting styles and interpersonal relationships. Front Psychol. 2024 [cited 2025 Dec 22];15. Available from: https://pubmed.ncbi.nlm.nih.gov/39539306/. 10.3389/fpsyg.2024.1477221 PMC11557463 39539306

[20] Buta BO, Mota ACP, Couto VVD, Tabak BM. Mental health literacy for public employees. BMC Public Health. 2024 Dec 1 [cited 2025 Dec 22];24(1). Available from: https://pubmed.ncbi.nlm.nih.gov/39334112/. 10.1186/s12889-024-19937-1 PMC11429317 39334112

[21] Chong SA, Abdin E, Nan L, Vaingankar JA, Subramaniam M. Prevalence and impact of mental and physical comorbidity in the adult Singapore population. Ann Acad Med Singap. 2012;41(3):105–14. 22538737

[22] Subramaniam M, Chong SA, Vaingankar JA, Abdin E, Chua BY, Chua HC, et al. Prevalence of dementia in people aged 60 years and above: results from the WiSE study. J Alzheimers Dis. 2015 [cited 2025 Dec 22];45(4):1127–38. Available from: https://pubmed.ncbi.nlm.nih.gov/25672767/. 10.3233/JAD-142769 25672767

[23] Subramaniam M, Abdin E, Vaingankar JA, Shafie S, Chua BY, Sambasivam R, et al. Tracking the mental health of a nation: prevalence and correlates of mental disorders in the second Singapore mental health study. Epidemiol Psychiatr Sci. 2020 [cited 2025 Jul 3];29. Available from: https://pubmed.ncbi.nlm.nih.gov/30947763/. 10.1017/S2045796019000179 PMC8061188 30947763

[24] Subramaniam M, Abdin E, Asharani PV, Roystonn K, Devi F, Peizhi W, et al. Prevalence of dementia in Singapore: Changes across a decade. Alzheimer’s and Dementia. 2025 Feb 1 [cited 2025 Jun 21];21(2). Available from: https://pubmed.ncbi.nlm.nih.gov/39868432/. 10.1002/alz.14485 PMC11848337 39868432

[25] Chong SA, Abdin E, Sherbourne C, Vaingankar J, Heng D, Yap M, et al. Treatment gap in common mental disorders: the Singapore perspective. Epidemiol Psychiatr Sci. 2012 Jun [cited 2025 Dec 22];21(2):195–202. Available from: https://www.cambridge.org/core/journals/epidemiology-and-psychiatric-sciences/article/abs/treatment-gap-in-common-mental-disorders-the-singapore-perspective/A5389F32B49AC65C2A4E1794C8F99D44. 10.1017/S2045796011000771 22789169

[26] Subramaniam M, Shahwan S, Abdin E, Tan YB, Gunasekaran S, Tan BCW, et al. Public stigma towards people with mental disorders in Singapore - Has anything changed? Asian J Psychiatr. 2026 Jan 1 [cited 2025 Dec 22];115. Available from: https://pubmed.ncbi.nlm.nih.gov/41270606/. 10.1016/j.ajp.2025.104771 41270606

[27] Tan R, Tay EH, Shahwan S, Tan YB, Gunasekaran S, Tan BCW, et al. Recognition and stigma of gambling disorder in Singapore. J Gambl Stud. 2025 Nov 18 [cited 2025 Dec 22];1–20. Available from: https://link.springer.com/article/10.1007/s10899-025-10457-0. 10.1007/s10899-025-10457-0 PMC13009059 41252045

[28] Tan YB, Tay EH, Shahwan S, Gunasekaran S, Lim BWZ, Tan BCW, et al. Factor structure and predictors of causal beliefs about seven mental illnesses among the Singapore general population. Front Public Health. 2025 [cited 2025 Dec 22];13:1612820. Available from: https://pmc.ncbi.nlm.nih.gov/articles/PMC12283601/. 10.3389/fpubh.2025.1612820 PMC12283601 40703187

[29] Schomerus G, Schwahn C, Holzinger A, Corrigan PW, Grabe HJ, Carta MG, et al. Evolution of public attitudes about mental illness: A systematic review and meta-analysis. Acta Psychiatr Scand. 2012 Jun [cited 2025 Jun 21];125(6):440–52. Available from: https://pubmed.ncbi.nlm.nih.gov/22242976/. 10.1111/j.1600-0447.2012.01826.x 22242976

[30] Angermeyer MC, Carta MG, Holzinger A, Matschinger H. The diffusion of the diagnostic term bipolar disorder among the German public. Psychiatry Res. 2018 Feb 1 [cited 2025 Jun 21];260:75–7. Available from: https://www.sciencedirect.com/science/article/abs/pii/S0165178117315020. 10.1016/j.psychres.2017.11.047 29175502

[31] Furnham A, Swami V. Mental health literacy: A review of what it is and why it matters. Int Perspect Psychol. 2018;7(4):240–57.

[32] Loosen AM, Skvortsova V, Hauser TU. Obsessive–compulsive symptoms and information seeking during the Covid-19 pandemic. Transl Psychiatry. 2021 Jun 1 [cited 2025 Jun 21];11(1):1–10. Available from: https://www.nature.com/articles/s41398-021-01410-x. 10.1038/s41398-021-01410-x PMC8138954 34021112

[33] Pavelko RL, Myrick JG. That’s so OCD: The effects of disease trivialization via social media on user perceptions and impression formation. Comput Human Behav. 2015 [cited 2025 Jun 21];49:251–8. Available from: https://pure.psu.edu/en/publications/thats-so-ocd-the-effects-of-disease-trivialization-via-social-med.

[34] Shahwan S, Goh CMJ, Tan GTH, Ong WJ, Chong SA, Subramaniam M. Strategies to reduce mental illness stigma: perspectives of people with lived experience and caregivers. Int J Environ Res Public Health. 2022 Feb 1 [cited 2025 Jun 21];19(3):1632. Available from: https://pmc.ncbi.nlm.nih.gov/articles/PMC8835394/. 10.3390/ijerph19031632 PMC8835394 35162655

[35] Reavley NJ, Jorm AF. Recognition of mental disorders and beliefs about treatment and outcome: Findings from an Australian National Survey of Mental Health Literacy and Stigma. Aust N Z J Psychiatry. 2011 Nov [cited 2025 Jun 21];45(11):947–56. Available from: https://pubmed.ncbi.nlm.nih.gov/21995330/. 10.3109/00048674.2011.621060 21995330

[36] Chen S, Wu Q, Qi C, Deng H, Wang X, He H, et al. Mental health literacy about schizophrenia and depression: A survey among Chinese caregivers of patients with mental disorder. BMC Psychiatry. 2017 Mar 9 [cited 2025 Jun 21];17(1):1–8. Available from: https://bmcpsychiatry.biomedcentral.com/articles/10.1186/s12888-017-1245-y. 10.1186/s12888-017-1245-y PMC5343538 28274209

[37] Holmberg C. Schizophrenia in print, digital, and audiovisual media: trends, topics, and results from an anti-stigma intervention targeting media professionals. Schizophr Bull Open. 2023 Jan 1 [cited 2025 Jun 21];4(1). Available from: 10.1093/schizbullopen/sgad018. 10.1093/schizbullopen/sgad018 PMC11207655 39145329

[38] Gwarjanski AR, Parrott S. Schizophrenia in the news: the role of news frames in shaping online reader dialogue about mental illness. Health Commun. 2018 Aug 3 [cited 2025 Jun 21];33(8):954–61. Available from: https://www.tandfonline.com/doi/abs/10.1080/10410236.2017.1323320. 10.1080/10410236.2017.1323320 28537757

[39] Murwasuminar B, Munro I, Recoche K. Mental health recovery for people with schizophrenia in Southeast Asia: A systematic review. J Psychiatr Ment Health Nurs. 2023 Aug 1 [cited 2025 Jun 21];30(4):620–36. Available from: https://pubmed.ncbi.nlm.nih.gov/36681884/. 10.1111/jpm.12902 36681884

[40] Yang LH, Lo G, WonPat-Borja AJ, Singla DR, Link BG, Phillips MR. Effects of labeling and interpersonal contact upon attitudes towards schizophrenia: implications for reducing mental illness stigma in urban China. Soc Psychiatry Psychiatr Epidemiol. 2011 Sep [cited 2025 Dec 22];47(9):1459. Available from: https://pmc.ncbi.nlm.nih.gov/articles/PMC3697873/. 10.1007/s00127-011-0452-y PMC3697873 22075964

[41] Howe L, Tickle A, Brown I. ‘Schizophrenia is a dirty word’: service users’ experiences of receiving a diagnosis of schizophrenia. The Psychiatric Bulletin. 2014 Aug [cited 2025 Dec 22];38(4):154. Available from: https://pmc.ncbi.nlm.nih.gov/articles/PMC4115437/. 10.1192/pb.bp.113.045179 PMC4115437 25237536

[42] Farrer L, Leach L, Griffiths KM, Christensen H, Jorm AF. Age differences in mental health literacy. BMC Public Health. 2008 [cited 2025 Jun 21];8. Available from: https://pubmed.ncbi.nlm.nih.gov/18423049/. 10.1186/1471-2458-8-125 PMC2358892 18423049

[43] Zhang H, Loi SM, Zhou S, Zhao M, Lv X, Wang J, et al. Dementia literacy among community-dwelling older adults in Urban China: a cross-sectional study. Front Public Health. 2017 Jun 7 [cited 2025 Jun 21];5. Available from: https://pubmed.ncbi.nlm.nih.gov/28638820/. 10.3389/fpubh.2017.00124 PMC5461251 28638820

[44] Zheng Z. Caregiving in Singapore. Stat Singap Newsl. 2011;12–4.

[45] Sahadevan S, Saw SM, Gao W, Tan LCS, Chin JJ, Hong CY, et al. Ethnic differences in Singapore’s dementia prevalence: The stroke, Parkinson’s disease, epilepsy, and dementia in Singapore study. J Am Geriatr Soc. 2008 Nov [cited 2025 Jun 21];56(11):2061–8. Available from: https://pubmed.ncbi.nlm.nih.gov/19016940/. 10.1111/j.1532-5415.2008.01992.x 19016940

[46] Pacifico D, Fiordelli M, Fadda M, Serena S, Piumatti G, Carlevaro F, et al. Dementia is (not) a natural part of ageing: a cross-sectional study on dementia knowledge and misconceptions in Swiss and Italian young adults, adults, and older adults. BMC Public Health. 2022 Dec 1 [cited 2025 Jun 21];22(1). Available from: https://pubmed.ncbi.nlm.nih.gov/36434540/. 10.1186/s12889-022-14578-8 PMC9701025 36434540

[47] De Vugt ME, Verhey FRJ. The impact of early dementia diagnosis and intervention on informal caregivers. Prog Neurobiol. 2013 Nov [cited 2025 Jun 21];110:54–62. Available from: https://pubmed.ncbi.nlm.nih.gov/23689068/. 10.1016/j.pneurobio.2013.04.005 23689068

[48] Howard M, McMillen C, Nower L, Elze D, Edmond T, Bricout J. Denial in addiction: Toward an integrated stage and process model—qualitative findings. J Psychoactive Drugs. 2002 [cited 2025 Jun 21];34(4):371–82. Available from: https://pubmed.ncbi.nlm.nih.gov/12562105/. 10.1080/02791072.2002.10399978 12562105

[49] Scott SB, Rhoades GK, Stanley SM, Allen ES, Markman HJ. Reasons for divorce and recollections of premarital intervention: implications for improving relationship education. Couple Family Psychol. 2013 [cited 2025 Jun 21];2(2):131. Available from: https://pmc.ncbi.nlm.nih.gov/articles/PMC4012696/. 10.1037/a0032025 PMC4012696 24818068

[50] O’Farrell TJ, Clements K. Review of outcome research on marital and family therapy in treatment for alcoholism. J Marital Fam Ther. 2012 Jan [cited 2025 Jun 21];38(1):122–44. Available from: https://pubmed.ncbi.nlm.nih.gov/22283384/. 10.1111/j.1752-0606.2011.00242.x PMC3270890 22283384

[51] Subramaniam M, Abdin E, Vaingankar JA, Shafie S, Chua BY, Sambasivam R, et al. Tracking the mental health of a nation: prevalence and correlates of mental disorders in the second Singapore mental health study. Epidemiol Psychiatr Sci. 2020 [cited 2025 Jun 21];29:e29. Available from: https://www.cambridge.org/core/journals/epidemiology-and-psychiatric-sciences/article/tracking-the-mental-health-of-a-nation-prevalence-and-correlates-of-mental-disorders-in-the-second-singapore-mental-health-study/91EF53CE124C3F7458D32700CCA08B8B. 10.1017/S2045796019000179 PMC8061188 30947763

[52] Lim WY, Subramaniam M, Abdin E, He VY, Vaingankar J, Chong SA. Lifetime and twelve-month prevalence of heavy-drinking in Singapore: Results from a representative cross-sectional study. BMC Public Health. 2013 Oct 21 [cited 2025 Jun 21];13(1). Available from: https://pubmed.ncbi.nlm.nih.gov/24499269/. 10.1186/1471-2458-13-992 PMC4028979 24499269

[53] Stewart E, Grunthal B, Collins L, Coles M. Public recognition and perceptions of obsessive compulsive disorder. Community Ment Health J. 2019 Jan 31 [cited 2025 Dec 22];55(1):74–82. Available from: https://pubmed.ncbi.nlm.nih.gov/30101380/. 10.1007/s10597-018-0323-z 30101380

[54] Syafeeq S, The Straits T. 2023 [cited 2025 Jun 21]. Research programme in S’pore to detect early depression in seniors through voice analysis | The Straits Times. Available from: https://www.straitstimes.com/singapore/research-programme-in-s-pore-to-detect-early-depression-in-seniors-through-voice-analysis.

[55] Teo J, The Straits T. 2024 [cited 2025 Jun 21]. S’pore launches national mental health and well-being strategy | The Straits Times. Available from: https://www.straitstimes.com/singapore/health/s-pore-launches-national-mental-health-and-well-being-strategy.

[56] Allport G. The nature of prejudice. Reading, MA: Addison-Wesley; 1954.

[57] Beainy C, Haddad C, Fekih-Romdhane F, Hallit S, Haddad G. Decreased insight, but not self-stigma or belief about medicine, is associated with greater severity of delusions in a sample of long-stay patients with schizophrenia: a cross-sectional study. BMC Psychiatry. 2023 Dec 1 [cited 2025 Jun 21];23(1). Available from: https://pubmed.ncbi.nlm.nih.gov/37013492/. 10.1186/s12888-023-04711-1 PMC10069113 37013492

[58] Department of Statistics Singapore. DOS | SingStat Website - National Accounts. 2025 [cited 2025 Dec 22]. Available from: https://www.singstat.gov.sg/publications/reference/ebook/economy/national-accounts.

[59] Shahwan S, Lau JH, Goh CMJ, Ong WJ, Tan GTH, Kwok KW, et al. The potential impact of an anti-stigma intervention on mental health help-seeking attitudes among university students. BMC Psychiatry. 2020 Dec 1 [cited 2025 Jun 21];20(1):1–14. Available from: https://bmcpsychiatry.biomedcentral.com/articles/10.1186/s12888-020-02960-y. 10.1186/s12888-020-02960-y PMC7690018 33238951

[60] Evans-Lacko S, London J, Japhet S, Rüsch N, Flach C, Corker E, et al. Mass social contact interventions and their effect on mental health related stigma and intended discrimination. BMC Public Health. 2012 Jun 28 [cited 2025 Jun 21];12(1):1–8. Available from: https://bmcpublichealth.biomedcentral.com/articles/10.1186/1471-2458-12-489. 10.1186/1471-2458-12-489 PMC3461459 22742085

[61] Gero K, Backhaus-Hoven I, Höhmann A, Dragano N, Hoven H. Low income and health literacy: a systematic scoping review. Arch Public Health. 2025 Nov 25 [cited 2025 Dec 22];83(1). Available from: https://pubmed.ncbi.nlm.nih.gov/41291950/. 10.1186/s13690-025-01781-3 PMC12667132 41291950

[62] Department of Statistics Singapore. DOS | SingStat Website - Education and Literacy. 2024 [cited 2025 Jun 21]. Available from: https://www.singstat.gov.sg/publications/reference/ebook/population/education-and-literacy.

[63] Ang HM, Channel News A. 2025 [cited 2025 Jun 21]. Median monthly household income exceeds S$11,000 in 2024, a 1.4% rise after adjusting for inflation - CNA. Available from: https://www.channelnewsasia.com/singapore/median-monthly-household-income-real-after-inflation-household-singstat-4934851.

[64] Andou J, Kitamura T, Andou J, Kitamura T. Gender differences in recognising depression in a case vignette in a university student population: Interaction of participant and vignette subject gender with depressive symptomatology. Open J Psychiatr. 2013 Oct 14 [cited 2025 Dec 22];3(4):384–92. Available from: https://www.scirp.org/journal/paperinformation?paperid=37859.

[65] Townsend L, Musci R, Stuart E, Heley K, Beaudry MB, Schweizer B, et al. Gender differences in depression literacy and stigma after a randomized controlled evaluation of a universal depression education program. J Adolesc Health. 2019 Apr 1 [cited 2025 Dec 22];64(4):472–7. Available from: https://pubmed.ncbi.nlm.nih.gov/30612807/. 10.1016/j.jadohealth.2018.10.298 PMC6571527 30612807

[66] Tan CM, Tay EH, Shahwan S, Tan YB, Gunasekaran S, Tan BCW, et al. Evolving perceptions of treatment helpfulness across mental illnesses in Singapore: 8-year comparison using nationally representative samples. BJPsych Open. 2025 Nov [cited 2025 Dec 22];11(6). Available from: https://pubmed.ncbi.nlm.nih.gov/41208364/. 10.1192/bjo.2025.10891 PMC12641415 41208364

[67] Kutcher S, Wei Y, Coniglio C. Mental health literacy: Past, present, and future. Can J Psychiatry. 2016 Mar 1 [cited 2025 Jun 21];61(3):154–8. Available from: https://pubmed.ncbi.nlm.nih.gov/27254090/. 10.1177/0706743715616609 PMC4813415 27254090

